# Epidermal Growth Factor Receptor (EGFR) Crosstalks in Liver Cancer

**DOI:** 10.3390/cancers3022444

**Published:** 2011-05-18

**Authors:** Carmen Berasain, María Ujue Latasa, Raquel Urtasun, Saioa Goñi, María Elizalde, Oihane Garcia-Irigoyen, María Azcona, Jesús Prieto, Matías A. Ávila

**Affiliations:** 1 Division of Hepatology and Gene Therapy, CIMA, University of Navarra, Pamplona 31008, Spain; E-Mails: mulatasa@unav.es (M.U.L.); rurtasun@unav.es (R.U.); sgirigoyen@alumni.unav.es (S.G.); melizalde.1@alumni.unav.es (M.E.); oihane.20@hotmail.com (O.G.-I.); mazcona@unav.es (M.A.); jprieto@unav.es (J.P.), maavila@unav.es (M.A.A.); 2. CIBERehd, University Clinic, University of Navarra, Pamplona 31080, Spain

**Keywords:** G protein-coupled receptor (GPCR), a desintegrin and metalloprotease (ADAM), transactivation, growth factor receptor

## Abstract

Hepatocarcinogenesis is a complex multistep process in which many different molecular pathways have been implicated. Hepatocellular carcinoma (HCC) is refractory to conventional chemotherapeutic agents, and the new targeted therapies are meeting with limited success. Interreceptor crosstalk and the positive feedback between different signaling systems are emerging as mechanisms of targeted therapy resistance. The identification of such interactions is therefore of particular relevance to improve therapeutic efficacy. Among the different signaling pathways activated in hepatocarcinogenesis the epidermal growth factor receptor (EGFR) system plays a prominent role, being recognized as a “signaling hub” where different extracellular growth and survival signals converge. EGFR can be transactivated in response to multiple heterologous ligands through the physical interaction with multiple receptors, the activity of intracellular kinases or the shedding of EGFR-ligands. In this article we review the crosstalk between the EGFR and other signaling pathways that could be relevant to liver cancer development and treatment.

## 1. Introduction

Hepatocellular carcinoma (HCC) is the most common primary liver malignancy in adults [1]. Because of the lack of effective treatment options prognosis of HCC is very poor. The number of HCC-related deaths almost equals the number of cases being diagnosed each year (more than 560,000), and the five-year survival rate is below 9% [2]. In the last years the detailed characterization of critical molecular pathways implicated in the pathogenesis of HCC has uncovered therapeutic targets that are being explored for their effectiveness in the prevention and treatment of HCC [3,4].

In the majority (90%) of cases HCC is the late complication of a chronic liver disease characterized by sustained liver damage, inflammation and hepatocellular proliferation. Therefore chronic hepatitis and cirrhosis are regarded as pre-neoplastic conditions and the infections by hepatitis B (HBV) and hepatitis C (HCV) viruses, chronic alcohol abuse or genetic conditions such as hereditary hemochromatosis and a 1-antitrypsin deficiency are considered risks factors for HCC. Among the various etiological agents some differences relevant to the carcinogenesis process have been identified. However, alterations in key molecular pathways such as WNT/β-catenin, hepatocyte growth factor (HGF)/mesenchymal-epithelial transition factor (c-Met), vascular endothelial growth factor (VEGF), insulin like growth factor receptor (IGF1R) and epidermal growth factor receptor (EGFR) are common to HCC development [4-6].

The EGFR system plays an essential role in cell proliferation, survival and migration and its altered activity has been implicated in the development and growth of many tumors including HCC [7]. Accordingly, the overexpression of EGFR and some of its ligands have been correlated with more aggressive liver tumors and poor survival [8,9].

In the last years, interreceptor crosstalk has received significant attention as an essential element in understanding the increasingly complex signaling networks operating within normal and cancer cells. Accumulating evidences suggest that the EGFR system acts as a “signaling hub” where different extracellular growth and survival signals converge [10,11]. The activation of EGFR by heterologous ligands as a consequence of the primary activation of another receptor is named transactivation. The ligand-dependent transactivation of EGFR implicates the activity of the ADAM (a disintegrin and metalloprotease) family of transmembrane metalloproteases and the shedding of EGFR ligands [12]. This transactivation can be triggered by multiple G-protein coupled receptors (GPCRs), cytokine receptors, integrins and other tyrosine kinase receptors (TKRs) [13-15]. The ligand-independent transactivation of EGFR has also been described, and involves the physical interaction of EGFR with other receptors such as platelet-derived growth factor receptor (PDGFR) [16] or IGF1R [17]. In addition, GPCR-ligands [15,18] and cytokines such as growth hormone (GH) and prolactin (PRL) [19] are able to phosphorylate the EGFR in the absence of EGFR-ligand shedding upon the activation of Src and Janus tyrosine kinase 1 (Jak1), respectively. In many tumor cells these inter-receptor communications have been linked to the resistance to tyrosine kinase inhibitors [18].

EGFR represents a rational target for anti-tumor strategies, however anti-EGFR agents have shown no effective response in HCC patients [20,21]. The better understanding of the extensive crosstalk and positive feedbacks between the different signaling systems may allow the development of synergistic antitumor therapies with reduced toxicity. Indeed as mentioned before, EGFR transactivation may thus represent a new therapeutic target [15,22]. In this review we summarize the crosstalk between EGFR and other signaling pathways that could be relevant to liver cancer development and treatment.

## 2. The EGFR System

EGFR, also known as ErbB1/HER1, is a 170 kDa transmembrane glycoprotein that defines a family of tyrosine kinase receptors (TKRs) including ErbB2/HER2, ErbB3/HER3 and ErbB4/HER4 [23]. These receptors are characterized by an extracellular ligand-binding domain, a transmembrane domain and a cytoplasmic domain containing the tyrosine kinase region followed by a carboxy-terminal tail with tyrosine autophosphorylation sites. This cytoplasmic domain is highly conserved among the different members of the family, except in ErbB3 in which key amino acids have been substituted resulting in the ablation of the tyrosine kinase activity [24]. The extracellular ligand-binding domain contains two cysteine-rich regions and is less well conserved among the different ErbB proteins, consistent with their ligand-binding specificities. With the exception of ErbB2, for which no ligand has been identified, the ErbB receptors can be bound by a family of growth factors that include EGF, transforming growth factor-α (TGF-α), amphiregulin (AR), epiregulin (EREG), heparin-binding EGF (HB-EGF), betacellulin (BTC) and epigen (EPG). These agonists exhibit differences in receptor affinity and specificity. They also possess functional selectivity stimulating different outcomes from the same receptor depending on their affinity [25] or on the ligand-receptor conformation induced [26] which determines a specific pattern of Tyr phosphorylation.

Upon ligand binding the homo- and/or hetero-dimerization of the receptor are induced resulting in the activation of its intrinsic tyrosine kinase activity and the subsequent autophosphorylation and cross-phosphorylation of the Tyr residues present in its C-terminal tail. The phosphorylated Tyr residues are recognized by adapter and effector molecules containing Src homology-2 (SH2) and phosphotyrosine binding (PTB) domains. These molecules include Shc, Grb7, Grb2, Crk, phospholipase Cγ (PLCγ), the kinases Src and PI3K, the protein phosphatases SHP1 and SHP2, and the Cbl E3 ubiquitin ligase [22,26]. Other enzymes like phospholipase D (PLD) and the transcription factors STAT 1, 3 and 5 do not bind the receptors but are also activated upon ligand binding [27]. All these interactions trigger intracellular signaling pathways resulting in the activation of extracellular signal-regulated kinase (ERK), c-jun NH2-terminal kinase (JNK) and p38 mitogen-activated protein kinase (p38-MAPK) through the ras/raf/MEK/MAPK cascade, the protein kinase C (PKC) pathway, the PI3K/Akt pathway (which can lead to NF-κB activation), and the STAT pathway [15,23,27]. These pathways control different transcriptional programs that regulate the expression of genes involved in proliferation, survival, differentiation and migration.

The EGFR ligands are expressed as type I transmembrane precursor proteins characterized by the presence of an EGF-like domain that defines receptor-binding specificity, an immunoglobulin-like domain, a hydrophobic transmembrane domain and a hydrophilic cytoplasmic tail. Additional motifs that include glycosylation sites and heparin-binding domains are also present in AR and HB-EGF [28-31]. These membrane-anchored peptides can be biologically active through juxtacrine signaling. However, in response to many physiological and pharmacological stimuli, the extracellular domain is proteolytically cleaved by transmembrane metalloproteases of the ADAM family in a process known as “ectodomain shedding”. Then the released soluble growth factors activate EGFR in a paracrine or autocrine manner [30,31]. The ectodomain shedding process generates remnant cytosolic fragments named carboxy-terminal fragments (CTFs). Recent works have demonstrated intriguing functions of CTFs, adding a degree of complexity to the functional relevance of the EGF family of growth factors [30].

Among the different members of the ADAM family involved in the shedding of EGFR ligands, AD AM17 or TACE (TNFα-converting enzyme) plays a central role [32]. Although the mechanisms of activation of ADAMs are largely unknown, its regulation is an important step in the modulation of EGFR signaling. Most ADAMs contain proline-rich regions and phophorylation sites in their cytoplasmic domains. Their phosphorylation by several protein kinases including PKC, Src or even ERK, the elevation of cytosolic Ca^2+^ levels, or the production of reactive oxygen species (ROS), have been implicated in the activation of ADAMs [12]. As we will discuss later, ADAMs are important players in the ligand-dependent transactivation of EGFR by GPCRs and other TKRs.

## 3. The EGFR System and Liver Cancer

EGFR is highly expressed in adult hepatocytes and the EGFR system plays a central hepatoprotective and pro-regenerative role in the liver [22,33]. Mice with targeted ablation of EGFR or null for HB-EGF or AR show delayed regeneration after partial hepatectomy (PH) [33-35]. In addition, the expression of different EGFR-ligands such as TGF-α, HB-EGF, EREG or AR is induced in the liver shortly after PH and upon injury in several models of acute liver damage. Importantly in these models the administration of HB-EGF, EGF or AR protects the liver against damage [22,36-38].

*In vitro*, numerous studies performed in human HCC cell lines corroborate the relevance of EGFR signaling in the maintenance of the transformed phenotype of HCC cells. The use of specific siRNAs for AR demonstrates that the autocrine loop based on this growth factor plays a non-redundant role and participates in the proliferation, anchorage-independent growth, survival, resistance to cytotoxic drugs and tumorigenicity of HCC cells [39,40]. In addition, the treatment of HCC cells with EGFR-specific tyrosine kinase inhibitors or neutralizing antibodies induces cell cycle arrest and apoptosis and increases chemosensitivity [41,42].

Besides these observations in human samples, different animal models also support the implication of this pathway in the development of HCC. Transgenic mice overexpressing TGF-α or EGF show a high incidence of HCCs [43,44], while TGF-α null mice show an attenuated response to carcinogens [45]. Importantly, in a rat model of chemically induced liver cirrhosis and HCC the inhibition of EGFR with the tyrosine kinase inhibitor gefitinib demonstrated an antitumoral effect [46].

It has been suggested that the protracted activation of these survival and proliferative mechanisms in response to persistent damage may lead to neoplastic transformation [22]. Indeed, as mentioned above, the continuous activation of EGFR signaling is a hallmark of HCC and contributes to the proliferation, resistance to apoptosis and invasive behavior of HCC cells [5,22]. It is accepted that in contrast to other tumors the main cause for EGFR activation in HCC is not the existence of EGFR mutations [47]. However a recent study reports the detection of the mRNA for the constitutively activated EGFR variant EGFRvIII in the serum of 37% of patients with HCC [48]. Interestingly, the expression of this variant has been related to enhanced tumorigenesis and resistance to cytostatic drugs in HCC cell lines [49]. In a recent report the use of specific anti-EGFRvIII antibodies has been proposed as a therapeutic strategy [50]. A mutation in the activation domain of ErbB2 has also been found in 11% of HCCs [51]. More frequently the activation of the EGFR pathway in HCC is associated with the overexpression of the receptors and/or the ligands creating autocrine/paracrine activation loops. The overexpression of EGFR or ErbB3, and of ErbB2 in HBV infected patients, is correlated with more aggressive tumors and poor survival [9,52,53]. The upregulation of TGF-α, HB-EGF or AR is detected not only in HCCs but also in preneoplastic conditions such as liver cirrhosis, suggesting a relevant role of this pathway from the early stages of hepatocarcinogenesis [29,34,46,54,55].

As mentioned above, ectodomain shedding is a crucial step in the control of EGFR-ligand availability and receptor activation. Interestingly, the expression of the metalloproteinase ADAM17/TACE is significantly induced in HCC and in the liver of cirrhotic patients [39,56] suggesting an increased availability of EGFR ligands from the early stages of hepatocarcinogenesis and reinforcing the implication of EGFR system in this process. The activity of these metalloproteinases is regulated in turn by multiple receptors and represents an essential step in the process of ligand-dependent EGFR transactivation. The interactions of EGFR with other receptors, its implication in the hepatocarcinogenesis process and its possible participation in the resistance to therapy are discussed in the next section.

## 4. EGFR Crosstalk

**Figure 1.**
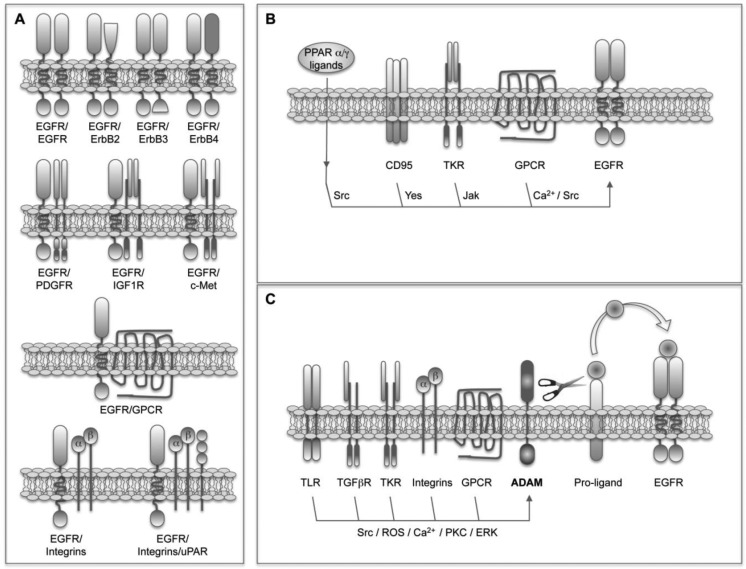
Epidermal growth factor receptor (EGFR) crosstalk. (**A**). EGFR physical interactions or heterodimerization with other membrane receptors. EGFR is able to bind to other receptors including other members of the ErbB family, TKRs, GPCRs and integrins; (**B**). Ligand-independent transactivation of EGFR; (**C**). Ligand-dependent transactivation of EGFR, or triple-membrane-passing signal (TMPS) pathway. Ligand-binding to TLRs, TGFpR, TKRs, integrins and GPCRs activates ADAM transmembrane metalloproteases resulting in the shedding of EGFR-ligands and the activation of EGFR.

The simplest and most obvious EGFR crosstalk involves its heterodimerization with other members of the family (Figure 1A). This process represents a way not only to amplify but also to diversify the signals [57]. It has been found that heterodimers trigger stronger signals than homodimers because of a faster receptor turnover [58]. In addition, as mentioned above, given the fact that the pattern of phosphorylated tyrosines and molecules recruited depends on each receptor, the selection of different dimerization partners will influence the outcome. For instance, the kinase-defective ErbB3 receptor induces an enhanced activation of the PI3K/Akt pathway because of the presence of multiple p85-binding sites [23], and the ligand-defective ErbB2 receptor potentiates signaling through the MAPK pathway due to reduced ligand dissociation rates [59]. Finally, it has been found that heterodimers acquire unique signaling properties that do not reflect simply the sum of the signals triggered by the individual partners [57]. All these facts together suggest that the repertoire of ErbB receptors expressed in a cell or tissue is essential to determine the outcome. As mentioned above, ErbB3 and ErbB2 are induced in HCC together with EGFR [52,53], in these conditions the formation of ErbB3/ErbB2, ErbB3/EGFR and ErbB2/EGFR heterodimers could be favored and could participate in transformation.

As mentioned before, the interplay between EGFR and other structurally distinct receptors places it as a signal integrator of multiple stimuli originated from the serum, nonmalignant stromal cells and the cancer cells themselves. This crosstalk takes place at a variety of levels and implies very different mechanisms including physical interactions, as well as ligand-dependent and ligand-independent transactivations (Figure 1).

The physical interaction or heterodimeratization of EGFR with other TKR receptors such as PDGFR [16,60], IGF1R [17] and c-MET [61], and with GPCRs such as somatostatin receptors 1 and 5 (SSTR) [62] have been reported (Figure 1A). The basal presence of PDGFβR-EGFR heterodimers has been demonstrated in different cell types where PDGF mediates the transactivation of EGFR upon ROS production and Src activation [16]. In those cells EGFR accounts for 30% of the PDGFR activity. The expression of PDGF is induced in the liver of cirrhotic patients and transgenic mice expressing human PDGF in the liver develop fibrosis and HCC [63]. However, the participation of EGFR in these events needs to be explored. The heterodimerization of EGFR with IGF1R in response to treatment with the EGFR TK inhibitor erlotinib has been described in non-small cell lung carcinoma (NSCLC) cells and is related to the subsequent development of drug resistance [17]. As we will discuss later, the crosstalk between EGFR and IGF1R also has important implications in the resistance of HCC cells to the EGFR-inhibitor gefinitib, however their physical interaction is not the mechanism of crosstalk in this case. The heterodimerization and crosstalk of EGFR with c-Met has also been related to EGFR-targeting drug resistance, and interfering with the HGF/c-Met pathway was proposed as a strategy to circumvent resistance to EGFR inhibitors [64]. It has been found that EGFR co-immunoprecipitates with c-Met in tumor cells, and this heterodimerization results in the EGFR-dependent activation of c-Met in the absence of HGF [61]. In HCC cells, however, this heterodimerization is not detected and the transactivation of c-Met upon EGFR activation occurs in a ROS-dependent manner [65]. The EGFR/c-Met interplay in liver cells is even more complex, as demonstrated by the fact that EGFR mediates the proliferation induced by HGF in cultured hepatocytes [66]. In breast cancer cells the interaction between the anti-proliferative GPCRs SSTR1 and SSTR5, and EGFR has been described [62]. Preformed heterodimeric SSTR/EGFR complexes exist that transmodulate EGFR function and dissociate in an EGFR-agonist-dependent manner. The inhibition of this dissociation by somatostatin (SST) attenuates the activation of downstream MAPK pathway [62]. Based on these interactions the use of SST analogues has emerged as a therapeutic strategy in cancer including HCC [67]. Another EGFR physical interaction described in osteosarcoma, breast and prostate cancer cells implicates the extranuclear steroid receptors androgen and estradiol receptors [68,69]. The effect and signaling of EGF in these cells depends on the association of estradiol receptor-α and androgen receptor with Src and EGFR, resulting in the steroid-independent regulation of EGFR [68]. The crosstalk between EGFR and integrins has been known from very long [70,71] and occurs at multiple levels including the activation of EGFR after physical interaction with α5β1-integrin [71,72]. Adding even more complexity to the system, it has been shown in head and neck tumor cells that the multiprotein complex formed by α5β1-integrin, the urokinase receptor (uPAR) and their ligands fibronectin (FN) and uPA, induces a high and persistent activation of ERK, which is accompanied by tumor cell proliferation after recruitment and activation of EGFR [73,74]. The upregulation of uPA and uPAR expression in HCC, and its relationship with the invasiveness, metastasis, and prognosis of HCC [75] suggest a possible role of the uPAR-integrin-EGFR-multiprotein complex in liver cancer.

In the absence of physical receptor interaction many GPCRs, cytokine receptors, nuclear hormone receptors and death receptors are able to induce ligand-independent EGFR transactivation (Figure 1B). In these cases EGFR is used as a scaffold protein. Specific residues in the cytoplasmic tail of EGFR are phosphorylated by non-receptor kinases such as Jak and Src providing docking sites for cytoplasmic signaling molecules. Different GPCR-ligands including angiotensin-II (ANG-II), prostaglandin E2 (PGE2), endothelin-1 (ET-1), IL-8, stromal cell-derived factor-1 (SDF-1) and beta2-adrenergic receptor-agonists mediate EGFR transactivation in a Ca^2+^-dependent or Src-dependent manner [10,76,77]. In human HCC cells, the crosstalk and positive feedback between cyclooxygenase-2 (COX-2)/PGE2 and EGFR has particular relevance and occurs at different levels. COX-2 is upregulated in HCC [78] and its product, PGE2, activates the GPCR EP1, inducing HCC cell proliferation and invasion in an EGFR-dependent Src-mediated manner [79]. Surprisingly, PGE2 also triggers the phosphorylation of c-Met in an EGFR-dependent manner [79] supporting the central role of EGFR in the coordination of signals relevant to HCC development. In addition, EGF induces the expression of COX-2 [79] and on the other hand PGE2 induces the expression of AR in HCC cells [34]. As we have already discussed and we will see later, GPCR-EGFR crosstalk occurs through different mechanisms and can be even bi-directional. Indeed, it has been demonstrated that EGF *trans*-regulates opioid receptors through EGFR-mediated tyrosine phosphorylation and activation of GPCR kinase 2 (GRK2) [80]. Regarding the interplay with cytokine receptors the binding of GH and PRL to their receptors trans-modulates EGFR signaling at least through three different mechanisms. First, the tyrosine phosphorylation-dependent activation of EGFR by Jak2 [19]; second, the control of EGFR turnover by threonine phosphorylation [81], and third the induction of EGFR expression [82]. These effects have been confirmed in the liver of both GH knockout and GH transgenic mice [82]. The relevance of this crosstalk in the hepatocarcinogenesis process should be characterized given the fact that GH transgenic mice are more susceptible to HCC development [82] and the expression of GH and PRL receptors is increased in human HCC [83]. Again in liver cells, it has been shown that the nuclear hormone receptor PPARα and PPARγ-agonists induce the rapid and transient activation of ERK through Src-dependent EGFR transactivation [84]. The importance and meaning of this interaction need to be further analyzed *in vivo*, given the fact that PPARγ is considered a tumor suppressor gene and clinical trials using PPARγ-agonists in the treatment of cancer are in course [85]. The activity of EGFR can be modulated by post-translational modifications other than phosphorylation. It has been recently shown that a regulatory crosstalk between protein arginine methyltransferase 5 (PRMT5)-mediated Arg 1175 methylation and EGF-induced Tyr 1173 phosphorylation attenuates EGFR-triggered ERK activation [86]. The implications of this crosstalk and the regulation of PRMT5 expression in cancer need further studies. Another remarkable interplay, again described in liver cells, refers to the interaction between EGFR and the death receptor CD95. Depending on the liver cell type and the signaling context this crosstalk results in either liver cell proliferation or apoptotic cell death [87]. In summary, the pro-apoptotic CD95 ligand, hydrophobic bile acids and hyperosmolarity induce the Yes-mediated ligand-independent EGFR transactivation, and depending on the presence of a sustained JNK activation the association of the activated EGFR with CD95 [87].

The ligand-dependent EGFR transactivation involves the activity of ADAM metalloproteinases, mainly ADAM17/TACE and ADAM10, and the shedding of EGFR ligands (Figure 1C). In a recent report it was demonstrated that a transforming Src mutant is able to stimulate ADAM17/TACE and increase the bioavailability of EGFR ligands [88]. Although the presence of Src mutants has not been described in liver cancer, c-Src activation is a common event in HCC [89,90] and could participate in the sustained activation of EGFR pathway implicated in hepatocarcinogenesis. Interestingly, in colon carcinoma cells and xenografts the activity of ADAM1 7/T ACE is induced in response to chemotherapy. The subsequent increased shedding of EGFR ligands and EGFR activation induce drug resistance [91]. However, in most of the cases the activation of ADAM17/TACE necessary to trigger a ligand-dependent EGFR transactivation depends on the previous binding of a heterologous agonist to its receptor. This process where the signal has to cross the cell membrane three times has been called triple-membrane-passing signal (TMPS) pathway [10]. Perhaps the best characterized TMPS pathway involves GPCR-agonists. In different cell types increased availability of EGFR ligands has been detected in response to ANG-II, lysophosphatidic acid (LPA), ET-1, thrombin, chemokines as IL-8 and CXCL12, Wnt and prostaglandins such as PGE2, among others [15,92-95]. Indeed it is accepted now that in many cases the unexpected mitogenic properties of certain neurotransmitters and hormones are dependent on EGFR transactivation. Different mechanisms have been proposed to mediate ADAM activation by GPCRs including the elevation of the intracellular levels of Ca^2+^ or ROS and the activation of kinases such as PKC, ERK, or c-Src [12]. TKRs can also participate in the TMPS pathway and this is the case for IGF1R [96,97]. Not in order Interestingly, in HCC cells the mitogenic effect of IGF2 requires the activation of EGFR induced upon release of AR [98]. The activation of ADAM17/TACE and the shedding of EGFR ligands have also been demonstrated after binding of TGFβ to its receptor in breast cancer cells [99] and hepatocytes [100] resulting in cell survival. As mentioned above the interaction between integrins and EGFR occurs at multiple levels. In HCC cells FN induces proliferation and invasion through the integrin/ADAM mediated transactivation of EGFR [101]. Another type of receptors implicated in TMPS is toll-like receptors (TLRs). In airway epithelial cells the release of IL8 and vascular endothelial growth factor (VEGF) induced in response to TLRs ligands depends on the activation of AD AM17/TACE, shedding of TGF-α and the activation of EGFR [102].

## 5. Therapeutic Implications of EGFR Crosstalk

The survival benefits of the tyrosine kinase inhibitor (TKI) sorafenib have demonstrated the utility of systemic targeted therapies for the treatment of advanced-stage HCC [103]. However, the molecular complexity and heterogeneity of HCC could be behind the moderate response to sorafenib treatment. In this regard, the identification of key molecules and pathways whose inhibition could enhance the efficacy of targeted therapeutic agents is essential. The central role played by EGFR in HCC biology underscores its potential as a therapeutic target, and different clinical trials have been undertaken using EGFR-specific TKIs and neutralizing antibodies alone [6] or more recently in combination with sorafenib [104].

In light of the observations summarized in this review, even in the absence of EGFR overexpression and enhanced shedding of EGFR ligands, EGFR crosstalk with other receptors that are activated in liver cancer reinforces its potential as a therapeutic target. On the other hand, the existence of this crosstalk could be also involved in the limited therapeutic efficacy of EGFR inhibitors in HCC [6]. In this regard, and as mentioned before, the crosstalk of EGFR with IGF1R [17] or c-Met [64] have been related to the resistance to EGFR inhibitors. Different phase 1 and 2 clinical trials for the treatment of advanced HCC are underway using targeted inhibitors for IGF1R (cixutumumab, AVE1642, BIIB022, IMC-A12 or OSI-906) and c-Met (ARQ197 or Foretinib) [104]. The combined therapy with EGFR and IGF1R or c-Met inhibitors could therefore be envisaged as a more effective strategy [64,97].

As we have extensively discussed, Src, ADAM and GPCRs have a central role in EGFR crosstalk. Src inhibitors such as AZD0530, dasatinib and bosutinib have been developed and tested in several clinical trials for the treatment of different types of solid tumors [105]. It would be worth evaluating the efficacy of these molecules in combination with EGFR inhibitors or sorafenib for the treatment of HCC. New orally bioavailable inhibitors of ADAM17/TACE have been developed [106]. The poor response to the EGFR inhibitor gefitinib in patients with non-small cell lung carcinoma was correlated with an increase in serum levels of AR and TGF-α [107]. We have shown that AR expression is induced in the liver of HCC patients [39] indicating that this resistance mechanism could be acting in HCC patients. The combined use of ADAM/TACE and EGFR inhibitors might thus be proposed to circumvent drug resistance. Regarding GPCRs, given their multiple interactions with EGFR, their role in hepatocarcinogenesis and the availability of specific inhibitors for some of them, different strategies can be conceived. As mentioned above, ET-1 transactivates EGFR in different ways [108]. Various clinical trials are underway to test the anticancer efficacy of the ET-1 receptor antagonists ZD4054 and atrasentan [109]. Given the fact that the levels of ET-1 are increased in the serum of HCC patients [110] testing the combination of ET-1 receptor antagonists and EGFR inhibitors for the treatment of HCC may be of interest. A similar combined strategy could be proposed for the inhibitor of the CXCL12 chemokine receptor CXCR4, BKT140. CXCL12 is known to transactivate EGFR [76], the axis CXCR4/CXCL12 is upregulated in HCC and participates in HCC cell proliferation [111] and BKT140 has been tested in clinical trials for the treatment of multiple myeloma [109]. Finally, as mentioned before, LPA transactivates EGFR [108] and its inductor, autotaxin, is significantly induced in HCC [112]. Palmitoyl a-bromomethylenephosphonate-1 (BrP-LPA) is an antagonist of LPA and also inhibits autotaxin [113], having demonstrated anticancer effects in a xenograft model of breast cancer [109]. These observations provide conceptual support for the evaluation of BrP-LPA as anticancer agent in HCC alone and in combination with EGFR targeting agents.

All in all, it could be expected that the combined inhibition of different partners participating in EGFR crosstalk might be more effective that the inhibition of a single event. Ideally, such strategies could also help to limit treatment toxicity by reducing the dose of individual agents, whereas the efficacy of the treatment would not be compromised due to the enhanced antitumoral effect of the combination.

## 6. Conclusions

The disruption of homeostasis, unrestrained proliferation and apoptosis resistance characteristic of cancer cells result from the alteration of many different interconnected pathways. The EGFR is considered as an important signaling hub where different proliferative and survival signals converge. This cooperative role of EGFR with other receptors in tumor growth could be exploited for therapeutic interventions. In this respect, the use of combined targeted therapies emerges as a promising alternative to overcome observed resistances.
